# The protective effects of Irbesartan in cognitive impairment in hypertension

**DOI:** 10.18632/aging.205589

**Published:** 2024-03-23

**Authors:** Shengyun Hao, Qian He, Yuan Yuan, Qiong Mu

**Affiliations:** 1 Department of General Medical, Affiliated Hospital of Guizhou Medical University, Guiyang 550001, China

**Keywords:** vascular cognitive impairment (VCI), Irbesartan, hypertension, cognitive impairment, cAMP/CREB

## Abstract

Vascular cognitive impairment (VCI) is claimed as the second most common type of dementia after Alzheimer’s disease (AD), in which hypertension is a critical inducer. Currently, hypertension-induced cognitive impairment lacks clinical treatments. Irbesartan is a long-acting angiotensin receptor antagonist with promising antihypertensive properties. Our research will focus on the potential function of Irbesartan on hypertension-induced cognitive impairment. Wistar-Kyoto (WKY) and spontaneously hypertensive (SHR) rats were orally dosed with normal saline or 20 mg/kg/day Irbesartan for 14 consecutive days, with 4 groups divided shown as below: WKY, Irbesartan, SHR, SHR+ Irbesartan. Firstly, the markedly increased systolic blood pressure observed in SHR rats was signally repressed by Irbesartan on Day 7 and 14 post-dosing. Moreover, notably decreased time of exploring the novel object in the object recognition task (ORT) test, elevated escape latency, and reduced time in the target quadrant in the Morris water maze (MWM) test were observed in SHR rats, which were prominently reversed by Irbesartan. Furthermore, the declined superoxide dismutase (SOD) activity, elevated malondialdehyde (MDA) level, increased cyclin-dependent kinase-5 (CDK5) activity, and enhanced protein level of p35/p25, p-Tau (pSer^214^)/Tau46, and brain-derived neurotrophic factor (BDNF) were memorably rescued by Irbesartan. Lastly, the activity of cAMP/cAMP response element binding protein (CREB) signaling in the hippocampus of SHR rats was markedly repressed, accompanied by an upregulation of phosphodiesterase 4B (PDE4B), which was observably rescued by Irbesartan. Collectively, Irbesartan protected against the hypertension-induced cognitive impairment in SHR rats by regulating the cAMP/CREB signaling.

## INTRODUCTION

Vascular cognitive impairment (VCI) refers to a group of diseases caused by vascular factors and characterized by cognitive impairment, ranging from mild VCI to vascular dementia [1]. VCI is considered to be the second most common type of dementia after Alzheimer’s disease (AD), accounting for about 15%-20% of the total number of dementia patients [2]. A domestic epidemiological study has shown that the prevalence of VCI in the Chinese population aged over 65 years was about 8.7%. The Canadian Study of Health and Aging showed that the incidence of mild VCI was higher than that of vascular dementia among respondents aged 65-84 years and that VCI patients had higher mortality and hospitalization rates [3]. Hypertension is reported to increase the risk of cognitive impairment and dementia [4]. The mechanisms of cognitive impairment caused by hypertension include excessive activation of the renin-angiotensin-aldosterone system (RAAS), oxidative stress (OS), endothelial dysfunction, inflammatory response, blood pressure variability, increased arterial stiffness, enlarged permeability of the blood-brain barrier, β-amyloid deposition, white matter arteriolar damage, brain atrophy, cerebral small vessel disease, and cerebral amyloid angiopathy [5]. It has been shown that angiotensin II enhances inflammatory response by activating leukocytes and facilitating the production of cell adhesion molecules, nicotinamide adenine dinucleotide phosphate hydrogen (NADPH) oxidase, pro-inflammatory cytokines, and reactive oxygen species (ROS). The production of ROS activates Toll-like receptors and triggers inflammatory responses, which in turn produces a cascade of reactions that induce OS. Progressive vascular injury disrupts the neurovascular unit (NVU), resulting in the destruction of the permeability of the blood-brain barrier, aggravating tissue hypoxia, and damaging neurons and white matter, which further contribute to cognitive decline [6, 7]. In addition, Guliaev et al. [8] reported that vasoconstriction was induced by the reduced production of nitric oxide (NO), a vasodilator factor, while vasoconstriction, together with endothelial cell shedding, dramatically aggravated the endothelial dysfunction, resulting in reduced cerebral blood flow and changes in the stability of blood-brain barrier, thereby causing ischemic damage of brain structure and cognitive impairment. Currently, the treatment strategies for hypertension-induced cognitive impairment are lacking in the clinic, which increases the attention and requests for developing novel agents to treat hypertension-induced cognitive impairment [9].

Irbesartan is a long-acting angiotensin receptor antagonist co-developed by Sanofi Saint-Drabbourg and Bristol-Myers Squibb. After approval on 30 September 1997, Irbesartan was launched in the United Kingdom, Germany, Italy, and Spain, successively [10, 11]. Irbesartan is a methyl biphenyltetrazolium angiotensin receptor blocker. Compared with other angiotensin receptor antagonist drugs, Irbesartan has the highest bioavailability with a plasma half-life of one hour, second only to telmisartan, which is a long-acting antihypertensive drug that can be taken daily [12]. Recently, several studies have revealed the anti-OS effects of Irbesartan in acute liver injury [13], renal tissues of type 2 diabetic rats [14], and angiotensin II treated endothelial cells [15]. However, the effects of Irbesartan on cognitive functions are less reported. In our research, the potential function of Irbesartan on hypertension-induced cognitive impairment will be explored.

## RESULTS

### Irbesartan repressed the hypertension in SHR rats

**Figure 1:**
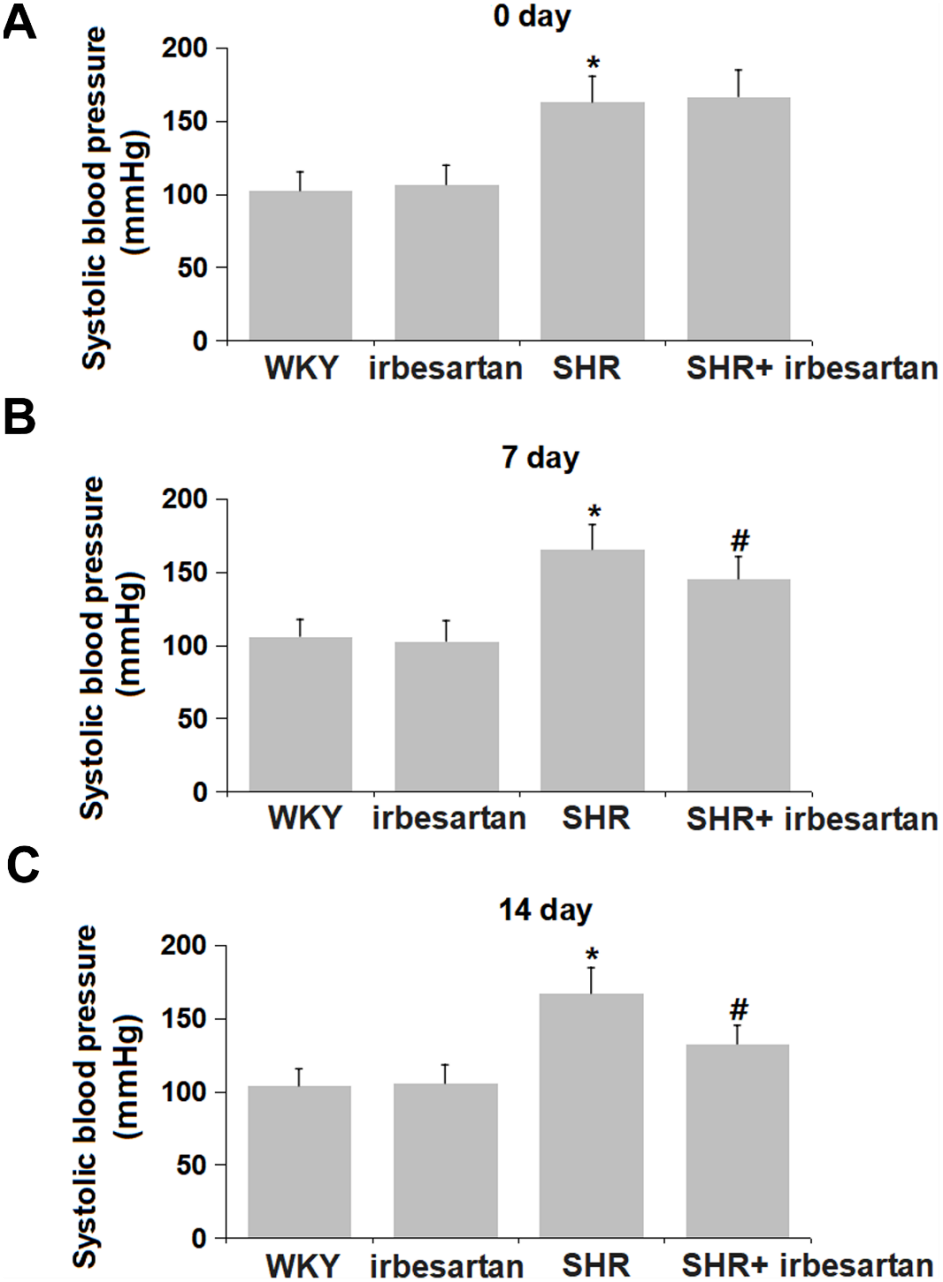
**Irbesartan repressed the hypertension in SHR rats.** (**A**) The systolic blood pressure on Day 0 post-dosing. (**B**) The systolic blood pressure on Day 7 post-dosing. (**C**) The systolic blood pressure on Day 14 post-dosing (n=6, *, P<0.005 vs. WKY group; #, P<0.05 vs. SHR group).

WKY rats and SHR rats were orally dosed with normal saline or 20 mg/kg/day Irbesartan for 14 consecutive days, during which the systolic blood pressure was monitored on Day 0, 7, and 14 post-dosing. On Day 0 post-dosing, the systolic blood pressure of WKY rats was kept around 105 mmHg, while the mean systolic blood pressure of SHR rats was dramatically increased to around 165 mmHg (Figure 1A). On Day 7 post-dosing, the systolic blood pressure in the WKY, Irbesartan, SHR, and SHR+ Irbesartan groups was 106.1, 102.8, 165.5, and 145.3 mmHg, respectively (Figure 1B). Furthermore, on Day 14 post-dosing, the systolic blood pressure was slightly changed from 103.8 to 105.6 mmHg in the Irbesartan, was signally elevated to 167.1 mmHg in the SHR group, then markedly reduced to 132.5 mmHg in the SHR+ Irbesartan group (Figure 1C). A remarkable suppressive property of Irbesartan against hypertension was observed in SHR rats.

### Effect of Irbesartan on ORT in SHR rats

**Figure 2:**
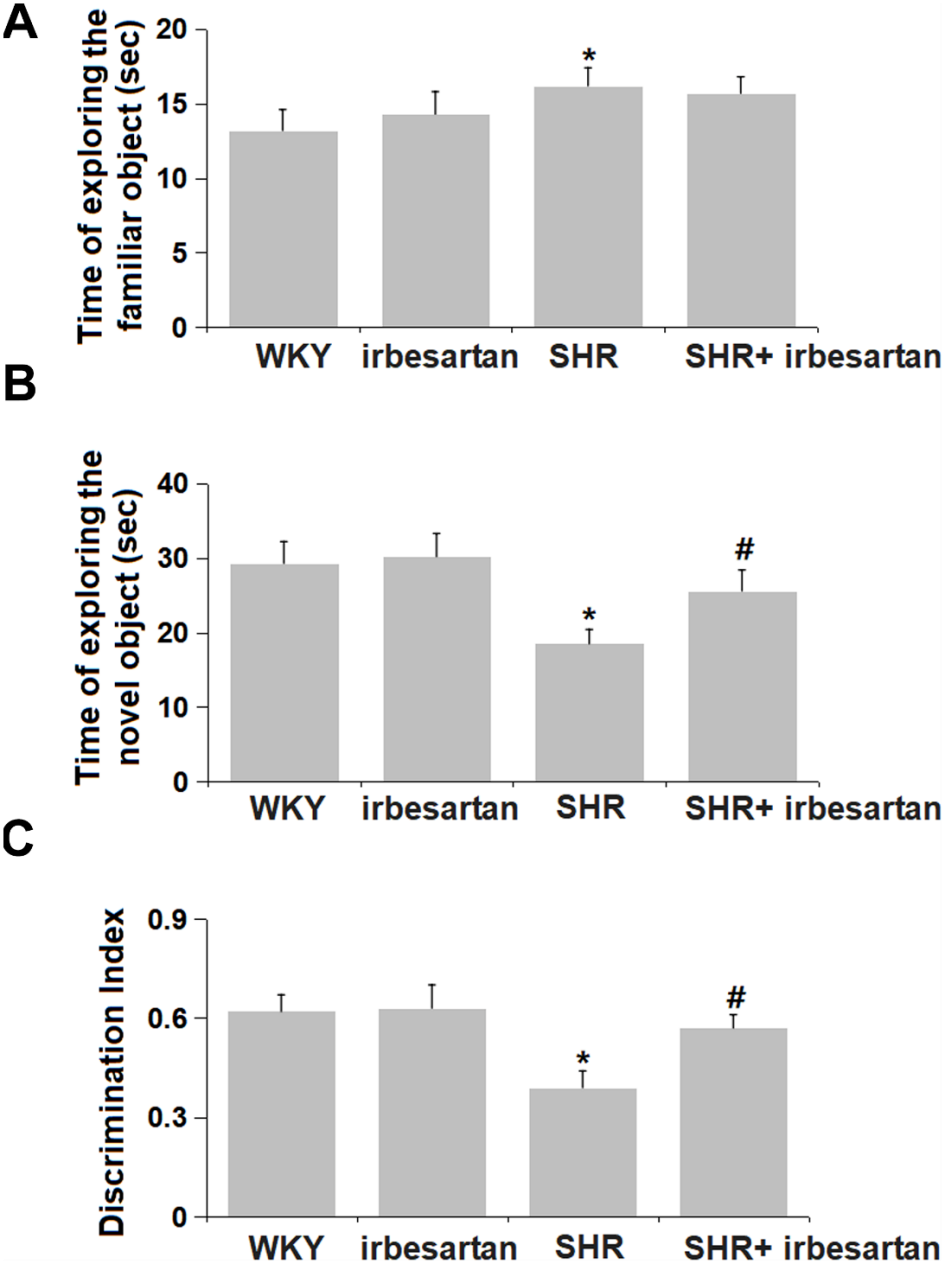
**Effect of Irbesartan on object recognition task (ORT) in SHR rats.** (**A**) Time of exploring the familiar object. (**B**) Time of exploring the novel object. (**C**) Discrimination Index (n=6, *, P<0.005 vs. WKY group; #, P<0.05 vs. SHR group).

ORT was performed on each rat for the evaluation of memory function. The time of exploring the familiar object in the WKY, Irbesartan, SHR, and SHR+ Irbesartan groups was 13.2, 14.3, 16.2, and 15.7 sec, respectively, with minor between-group differences (Figure 2A). However, the time of exploring the novel object was slightly changed from 29.3 sec to 30.2 sec in the Irbesartan group, was markedly declined to 18.5 sec in the SHR group, and then notably increased to 25.6 sec in the SHR+ Irbesartan group (Figure 2B). Furthermore, the discrimination index in the WKY, Irbesartan, SHR, and SHR+ Irbesartan groups was 0.62, 0.63, 0.39, and 0.57, respectively (Figure 2C). Irbesartan showed a promising protective effect against impaired memory function in SHR rats.

### Irbesartan alleviated spatial learning and memory in the MWM test in SHR rats

**Figure 3:**
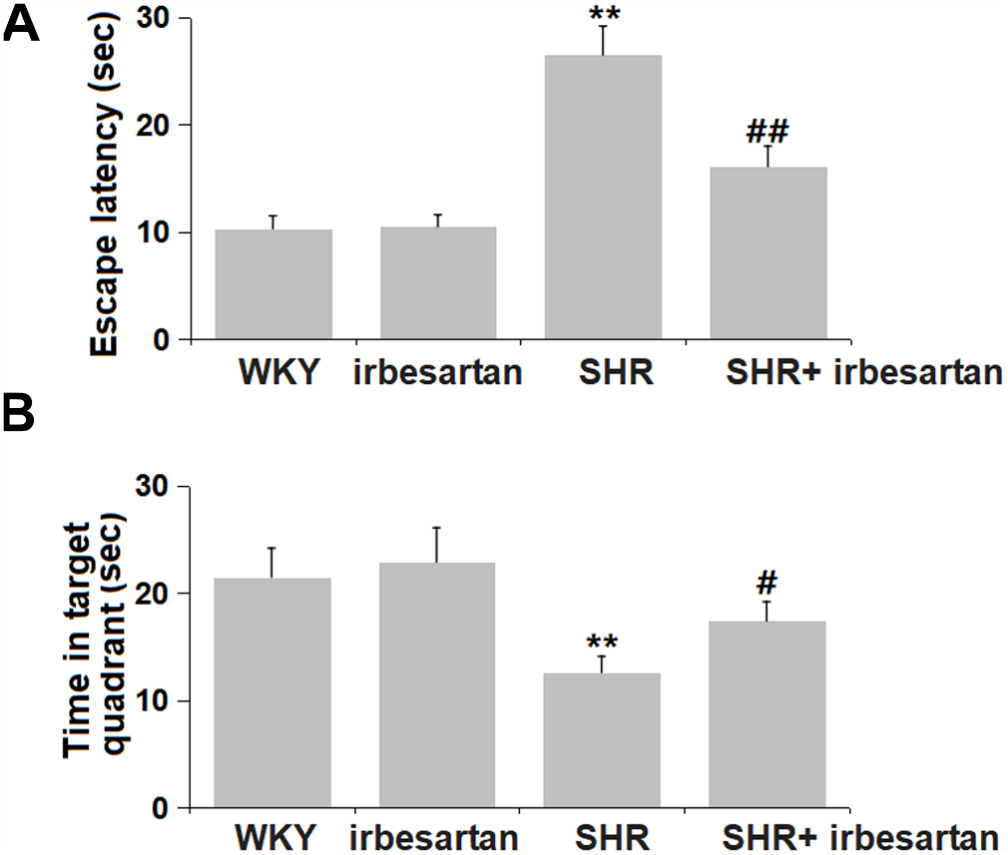
**Irbesartan alleviated spatial learning and memory in Morris water maze (MWM) test in SHR rats.** (**A**) Escape latency. (**B**) Time in target quadrant (n=6, **, P< 0.01 vs. WKY group; #, ##, P<0.05, 0.01 vs. SHR group).

To evaluate the impact of Irbesartan on learning and memory function in SHR rats, the MWM test was performed. The escape latency was slightly altered from 10.3 sec to 10.5 sec in the Irbesartan group, was markedly increased to 26.5 sec in the SHR group, then memorably reduced to 16.1 sec in the SHR+ Irbesartan group (Figure 3A). Furthermore, the time in the target quadrant in the WKY, Irbesartan, SHR, and SHR+ Irbesartan groups was 21.5, 22.9, 12.6, and 17.4 sec, respectively (Figure 3B). Irbesartan exerted an alleviative property against impaired spatial learning and memory in SHR rats.

### Irbesartan mitigated the OS in the hippocampus of SHR rats

**Figure 4:**
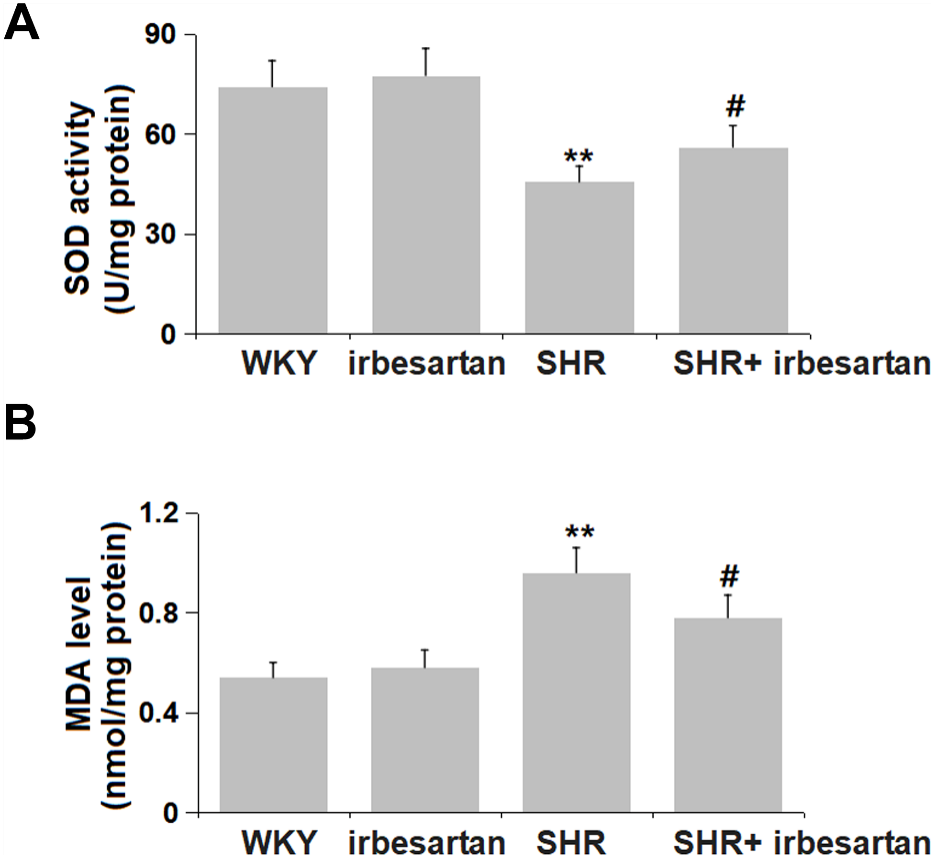
**Irbesartan mitigated the oxidative stress in the hippocampus of SHR rats.** (**A**) SOD activity and (**B**) MDA level were detected by commercial kits (n=6, **, P< 0.01 vs. WKY group; ##, P< 0.01 vs. SHR group).

OS is a critical factor involved in the progression of hypertension-induced cognitive impairment [16]. The SOD activity in the hippocampus was found slightly altered from 74.2 to 77.6 U/mg protein in the Irbesartan group, markedly reduced to 45.6 U/mg protein in the SHR group, then notably reversed to 56.1 U/mg protein by Irbesartan (Figure 4A). Moreover, the MDA level in the hippocampus in the WKY, Irbesartan, SHR, and SHR+ Irbesartan groups was 0.54, 0.58, 0.96, and 0.78 nmol/mg protein, respectively. A repressive property of Irbesartan against OS in the hippocampus was observed in SHR rats.

### Irbesartan repressed CDK5 activity and reversed BDNF reduction in the hippocampus of SHR rats

**Figure 5:**
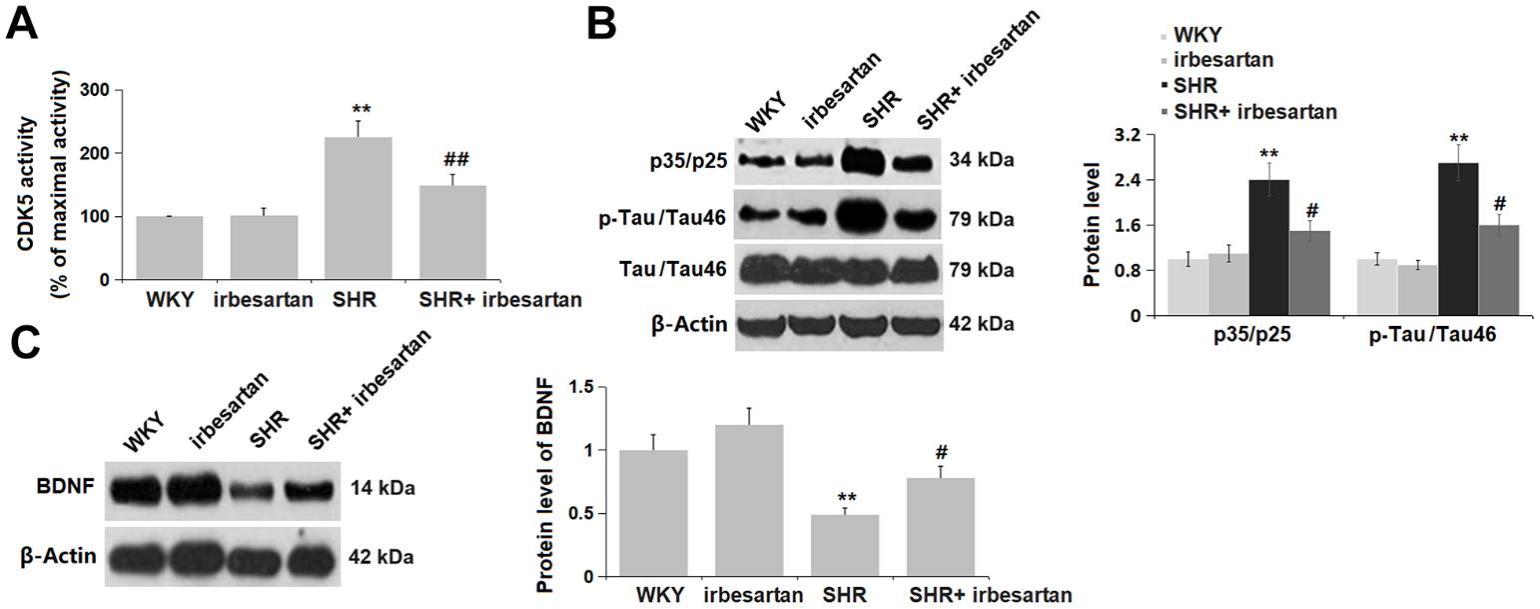
**Irbesartan repressed CDK5 activity and reversed BDNF reduction in the hippocampus of SHR rats.** (**A**) CDK5 activity (% of maximal activity) was determined by ELISA. (**B**) Protein level of p35/p25 and p-Tau (pSer^214^)/Tau46. (**C**) Protein level of BDNF(n=6,**, P< 0.01 vs. WKY group; #, ##, P<0.05, 0.01 vs. SHR group).

CDK5 activity [17] and BDNF levels [18] are critical biomarkers reflecting cognitive function. The CDK5 activity and protein levels of p35/p25 and p-Tau (pSer^214^)/Tau46 were slightly altered in the Irbesartan group, signally elevated in the SHR group, then markedly repressed in the SHR+ Irbesartan group (Figure 5A, 5B). Moreover, the BDNF level in the Irbesartan group was slightly changed, and was then observably decreased in the SHR group, which was memorably reversed by Irbesartan (Figure 5C). The CDK5 activity was found repressed, and the BDNF level increased by Irbesartan in the hippocampus of SHR rats.

### Irbesartan activated cAMP/CREB signaling in the hippocampus of SHR rats

**Figure 6:**
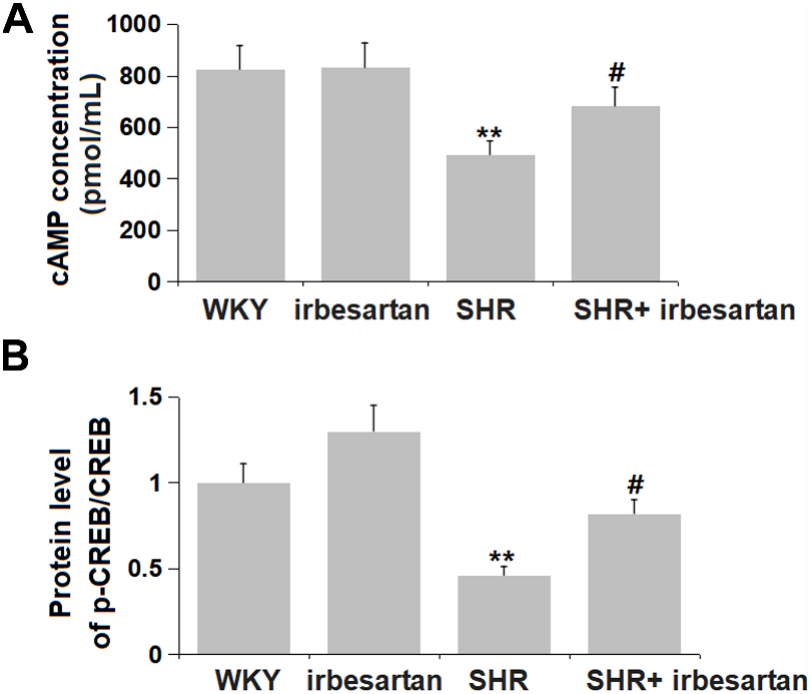
**Irbesartan activated cAMP/CREB signaling in the hippocampus of SHR rats.** (**A**) cAMP concentration was detected by ELISA. (**B**) Protein level of p-CREB/CREB(n=6, **, P<0.005, 0.01 vs. WKY group; #, P<0.05, vs. SHR group).

cAMP/CREB signaling is reported to mediate cognitive function during hypertension [19]. The cAMP concentration in the Irbesartan group was slightly changed from 823.9 to 831.1 pmol/mL, and was then markedly reduced to 492.5 pmol/mL in the SHR group, which was signally reversed to 681.5 pmol/mL by Irbesartan (Figure 6A). Moreover, the p-CREB/CREB level in the Irbesartan group was slightly altered, then observably repressed in the SHR group, which was signally reversed by Irbesartan (Figure 6B). cAMP/CREB signaling was found markedly activated by Irbesartan in the hippocampus of SHR rats.

### Irbesartan inhibited the increased PDE4B level in the hippocampus of SHR rats

**Figure 7:**
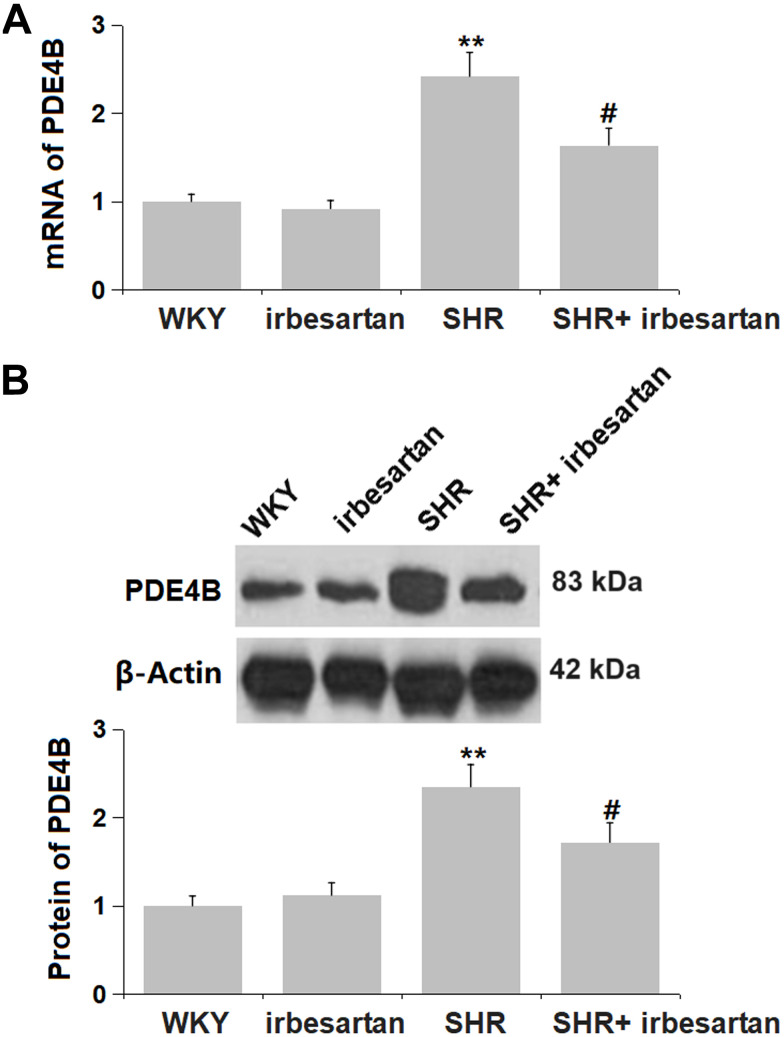
**Irbesartan inhibited the increased PDE4B level in the hippocampus of SHR rats.** (**A**) mRNA of PDE4B; (**B**) Protein of PDE4B(n=6, **, P<0.005, 0.01 vs. WKY group; #, P<0.05vs. SHR group).

PDE4B is a negative regulator for the activation of the cAMP/CREB signaling [20]. The mRNA and protein levels of PDE4B were found hardly changed in the Irbesartan group but markedly elevated in the SHR group, which was notably reversed in the SHR+ Irbesartan group (Figure 7A, 7B).

## DISCUSSION

Cognitive function refers to the ability of the human brain to process, store and extract information, including perception, memory, executive power, imagination, thinking and language [21]. Cognitive dysfunction is defined as the impairment of the brain’s ability to store, process and extract information, triggered by various inducers. Cognitive impairment includes stages ranging from mild cognitive impairment to dementia. COPERNICUS’s study [22, 23] focused on the elderly population and showed that hypertension in the elderly was associated with a higher risk of cognitive impairment. In our research, old SHR rats were used to investigate hypertension-induced cognitive impairment, with WKY rats taken as the negative control, widely applied by previous studies [24, 25]. In line with data presented by Yamada [26] and Lee [27], notably increased systolic blood pressure, decreased time of exploring the novel object in the ORT test, and elevated escape latency in the MWM test were observed in SHR rats. Following the administration of Irbesartan, the systolic blood pressure, time of exploring the novel object, and escape latency were markedly reversed in SHR rats, implying an alleviative property of Irbesartan on hypertension-induced memory and cognitive impairment in SHR rats.

The brain is mainly composed of lipids that are easily oxidized, with a high oxygen consumption rate, which lacks efficient oxidative defense mechanisms. Therefore, the brain is more sensitive to OS [28]. Chronic OS is considered to be a common pathological process of neurodegenerative diseases such as vascular dementia and AD, which can lead to persistent and progressive cognitive impairment by phosphorylation of tau protein in cortical neurons and deposition of Aβ immunoreaction products [29]. In our research, an activated OS was observed in the hippocampus of SHR rats, consistent with data reported by Kishi [30]. After the administration of Irbesartan, the state of OS in the hippocampus of SHR rats was markedly ameliorated, suggesting that the function of Irbesartan might be correlated with the inhibition on OS in the hippocampus. Furthermore, CDK activity, along with the downstream p35/p25 and p-Tau (pSer^214^)/Tau46 levels, were activated in the hippocampus of SHR rats, which were critical biomarkers of cognitive dysfunction reported by Corbel [31]. Following Irbesartan administration, the CDK signaling was markedly repressed, implying a potential correlation between the therapeutic function of Irbesartan and CDK signaling.

cAMP responsive element binding protein (CREB) is an important nuclear transcription factor that usually exists in the nucleus in an inactive form, while phosphorylated CREB (p-CREB) is active. CREB can be phosphorylated by different molecules at multiple sites, thereby regulating CREB transcription, participating in cell differentiation and regeneration, and regulating protein regeneration [32]. Studies have shown that CREB is an important factor in the formation of learning and memory [33, 34]. The abnormal expression of CREB has been confirmed to be related to the occurrence of central nervous system diseases, such as Alzheimer’s disease, epilepsy, and vascular dementia [35, 36] In our study, repressed cAMP/CREB signaling was observed in SHR rats, also claimed by Goel in LPS-treated SHR rats [37]. Following Irbesartan administration, cAMP/CREB signaling was markedly activated, implying that the function of Irbesartan might be correlated with the activation of cAMP/CREB signaling. The PDE superfamily are the only key enzyme known to hydrolyze cAMP in cells. PDE4B regulates the intracellular cAMP level by hydrolyzing it, thereby regulating its downstream pathways and producing corresponding effects [38]. In our study, the activating effect of Irbesartan on cAMP/CREB signaling in the hippocampus of SHR rats was accompanied by a downregulation of PDE4B, suggesting that the regulatory function of Irbesartan on cAMP/CREB signaling might be mediated by PDE4B. In future investigations, the regulatory mechanism will be further identified by administering Irbesartan into PDE4B-overexressed transgenic SHR rats.

In conclusion, our findings demonstrate that Irbesartan rescued the declined SOD activity and suppressed the MDA level, resulting in the attenuation of OS. Moreover, Irbesartan repressed the CDK activity and reduced the levels of p35/p25 and p-Tau (pSer^214^)/Tau46. Importantly, Irbesartan activated the cAMP/CREB signaling by inhibiting the expression of PDE4B at both the mRNA and protein levels. Collectively, Irbesartan protected against the hypertension-induced cognitive impairment in SHR rats mediated by the cAMP/CREB signaling. As a long-trusted therapy, Irbesartan treatment shows its potential benefits for hypertension-induced cognitive impairment. In the future, the mechanism underlying the protective effects of Irbesartan will be further elucidated to provide valuable evidence for its clinical implications in the treatment of VCI.

## MATERIALS AND METHODS

### Animals and grouping

24-week-old male spontaneously hypertensive rats (SHR) and Wistar-Kyoto (WKY) rats were obtained from Charles River (Beijing, China). The experiments were approved by the Animal Care Committee of “The Affiliated Hospital of Guizhou Medical University”.

After one week of adaption, WKY and SHR rats were orally dosed with normal saline or 20 mg/kg/day Irbesartan (#138402-11-6, Sigma-Aldrich, China) for 14 consecutive days, with four groups divided shown as below: WKY, Irbesartan, SHR, SHR+ Irbesartan.

### Measurement of the systolic blood pressure

On Day 0, 7, and 14 post posing, the systolic blood pressure of each animal was detected using a small animal blood pressure monitor (RWD Life Science, China).

### Object recognition task

Two brightly colored objects (similar in size, slightly different in color and shape) of A and B were placed in a rat cage. Due to curiosity about novel things, rats actively explore (smell, grasp, etc.) two objects. Rats in each group were explored for the first time and the time of exploring objects A and B within 10 minutes was recorded. After an interval of 48 h, object B was removed and replaced with object C (a novel object). The time of exploring objects A and C was recorded to determine the time of exploring the familiar object and the time of exploring the novel object, respectively. The discrimination index was determined as (C/A+C)×100%.

### Morris water maze (MWM) test

The maze consisted of a water tank (200 cm in diameter and 60 cm in height) filled with water and was divided into four quadrants, one of which had a concealed platform with a diameter of 10 cm that was located 1 cm below the water surface. The local navigation test was conducted for 5 days and 2 hours each day. The rat platform was placed in the center of any of the quadrants and the rat was allowed to enter the water from any of the other 3 quadrants facing the pool wall. The time that the rats found and climbed onto the platform was observed and recorded as the escape latency time. If the platform was not found within 60 s, the rat was guided to the platform for 20 s, and the escape latency time was recorded as 60 s. The space probe test was performed on the second day after the place navigation test was completed. The rat was dropped into the water twice from the opposite side of the original target quadrant and allowed to explore freely for 120 s. The number of times that the rat crossed the hidden platform and the trajectory was recorded using a video tracking system.

### The measurement of MDA level and SOD activity

A commercial kit (Qingdao Jisskang Biotechnology, China) was applied to measure the MDA level in the hippocampus with the TBA method. The SOD activity in the hippocampus was tested using a commercial kit (Beyotime, China) with the nitroblue tetrazolium (NBT) method. The instruction of the kits was strictly followed.

### ELISA

Wells were loaded with different concentrations of standards and testing samples, which were then introduced with 40 μL sample diluent, followed by loading horseradish peroxidase (HRP) labeled antibody. After sealing and incubating at 37° C for 60min in the incubator, the solution was removed with the absorbent paper and the washing liquid was added for washing and repeated 5 times, followed by introducing substrates A and B to be incubated for 15 min at 37° C in the dark. The OD value of each well was determined using a microplate reader (BioTek, USA) within 15 min after 50 μL of termination solution was added.

### Real-time polymerase chain reaction (PCR)

After obtaining RNAs from hippocampus tissues with the TRIzol reagent (Invitrogen, USA), the ultraviolet spectrophotometer (Hach, USA) was applied for the quantification of RNAs, followed by conducting the transcription to cDNAs utilizing a cDNA synthesis kit (SolelyBio, China). Then, PCR was performed with a SYBR Premix Ex TaqII kit (Takara, Japan), and the 2^−ΔΔCt^ method was applied for the calculation of gene levels. The following primers were used in the current study:

PDE4B:forward: 5’ -GACTGGTACTTCATGCCGCCTTC-3’,

reverse: 5’ -ATCAGGTCATCGCCGTGTTGTTC-3’;

β-actin: forward: 5’-ACAACCTTCTTGCAGCTCCTC-3’,

reverse: 5’-AGGATTCCATACCCAGGAAGG-3’.

### Western blot analysis

Total proteins from the hippocampus were extracted using the lysis and quantified using the bicinchoninic acid (BCA) (Sigma-Aldrich, USA) method. The separation of total proteins was conducted using the 12% sodium dodecyl sulphate-polyacrylamide gel electrophoresis (SDS-PAGE), while separated proteins were transferred to the polyvinylidene fluoride (PVDF) membranes (Invitrogen, USA). The membranes were incubated with 5% non-fat milk for 2 h. Then the membranes were treated with the antibodies against p-Tau (pSer^214^) (#ab170892, 1:500), Tau46 (#ab203179, 1:2000), BDNF (#ab108319, 1:3000), p-CREB (#ab32096, 1:500), CREB (#ab32515, 1:3000), PDE4B (#ab170939, 1:2000) and β-actin (#HC201-01, TransGen Biotech, China, 1:5000) were imported. Subsequently, the secondary antibody (#RGAM002, 1:2000, Proteintech, USA) was introduced. The exposure of bands was conducted with the ECL solution for quantification.

### Statistical analysis

The results are shown as Mean ± standard deviation (S.D.). All data were analyzed with the one-way analysis of variance (ANOVA), followed by Tukey’s post-hoc test. Statistical analysis was performed using the software GraphPad Prism 6.0. P< 0.05 was considered a statistically significant difference.

### Availability of data and materials

The data generated in the present study may be requested from the corresponding author.
